# Reduction-responsive supramolecular hybridized paclitaxel nanoparticles for tumor treatment

**DOI:** 10.3389/fbioe.2023.1257788

**Published:** 2023-09-01

**Authors:** Yuhan Wang, Yingli Cui, Tinggeng Dai, Ying Yue

**Affiliations:** Department of Gynecological Oncology, The First Hospital of Jilin University, Changchun, Jilin, China

**Keywords:** paclitaxel, organosilica, reduction-responsive, controlled drug release, chemotherapy

## Abstract

Powerful chemotherapeutics have been used to combat tumor cells, but serious adverse effects and poor therapeutic efficiency restrict their clinical performance. Herein, we developed reduction-responsive supramolecular hybridized paclitaxel nanoparticles (PTX@HOMNs) for improved tumor treatment. The nanocarrier is composed of F127 and strengthened by a disulfide bond linked organosilica network, which ensures the desirable stability during blood circulation and controlled drug release at tumor sites. The as-prepared PTX@HOMNs could effectively accumulate at tumor regions. After entering tumor cells, PTX@HOMNs can respond to intracellular glutathione, and trigger active drug release for chemotherapy. As a result, PTX@HOMNs exhibited potent antitumor activity against ovarian tumors *in vitro* and *in vivo*. Our work provides a deep insight into constructing simple and controlled drug delivery nanoplatforms for improved tumor treatment.

## 1 Introduction

Ovarian carcinoma is the most fatal gynaecological cancer with a poor prognosis (Lheureux et al., 2019; Menon et al., 2021). Chemotherapy is the mainstay treatment regimen in clinical practice for ovarian cancer treatment (Kuroki and Guntupalli, 2020; Blagden, 2022; Frenel et al., 2022). As one of the first-line chemotherapeutic drugs for many malignant tumors, paclitaxel (PTX) exhibits superior clinical performance (Yin et al., 2021; Clamp et al., 2022). PTX can promote the polymerization of β-tubulin subunits and microtubule assembly, and prevent depolymerization, thus leading to disordered cell mitosis and cell apoptosis (Horwitz, 1994; Alhussan and Chithrani, 2021). However, PTX has drawbacks of poor solubility, non-selective distribution, rapid pharmacokinetics, and drug resistance (Smith et al., 2022). Taxol^®^ uses Cremolphore EL and ethanol to formulate PTX to improve the solubility issues (Rowinsky and Donehower, 1995). The co-solvent brings about serious side effects, including hypersensitivity reactions, myelosuppression, and cumulative neurotoxicity (Zang et al., 2019; Li et al., 2020).

Various nanoparticle-based drug delivery systems have been designed and applied to address the above issues (Lang et al., 2019; Sun et al., 2019; Liu et al., 2022). The nanoparticles could improve drug availability through enhanced permeability and retention (EPR) effects (Ding et al., 2020; Fang et al., 2020; Takakura and Takahashi, 2022; Xu et al., 2023). As is known, poly(ethylene oxide) poly (propylene oxide)-poly(ethylene oxide) (PEO-PPO-PEO) triblock copolymers, Pluronic F127 has been approved by the Food and Drug Administration (FDA) as a pharmaceutical ingredient (Liu et al., 2021; Xia et al., 2021; Zhou et al., 2021). The F127-based drug delivery nanoplatforms have wined enormous development (Bhattacharya et al., 2020; Li et al., 2022; Park et al., 2022). These nanoformulations suffer from the problems of poor physiological stability and premature drug release during blood circulation (Bernabeu et al., 2017; Chou et al., 2020). Rationally designed controlled nanoparticles are imperative to guarantee a good therapeutic window. Tumor microenvironment overexpressed biomarkers, including acid, hypoxia, and redox, provide munch chance to build responsive nanosystems to enhance the tumor-specific cell-killing efficacy (Li et al., 2021; Lu et al., 2021; Xia et al., 2021; Hao et al., 2022; Hao et al., 2023; Lu et al., 2023). The reduction-responsive nanoparticles exhibited excellent promise in improving bioavailability, realizing the efficient use of drugs (An et al., 2023). With the characteristic of stimulus-response, the nanoparticles with minimum leakage could diminish toxic side effects in normal tissues and promote cellular uptake in tumor areas (Liu et al., 2022).

**SCHEME 1:**
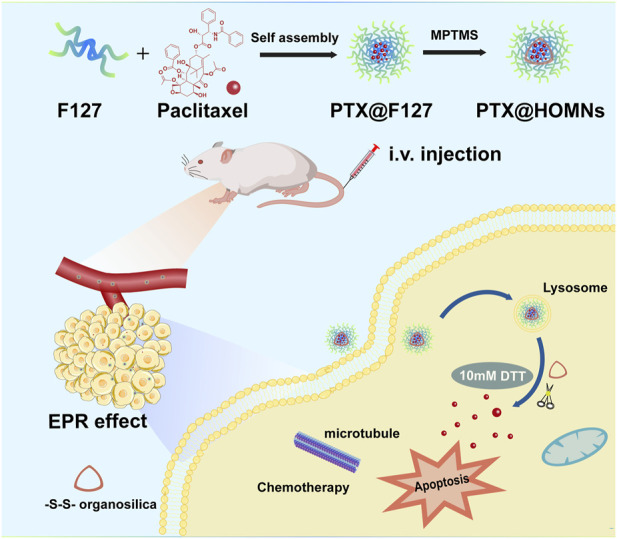
Schematic illustration of the preparation of PTX@HOMNs and their antitumor activity derived from reduction-responsive drug release.

Herein, we developed reduction-responsive supramolecular hybridized PTX nanoparticles (PTX@HOMNs) for combating ovarian carcinoma (Scheme 1). The PTX-loaded F127 micelles were post-modified with disulfide bond-linked organosilica. The introduction of organosilica not only improved colloidal stability and avoided undesirable drug leverage, but also controlled drug release at tumor sites, thus enhancing drug availability at tumor sites. PTX@HOMNs exhibit potent tumor suppression *in vitro* and *in vivo* and ignorable systemic toxicity. This investigation provides a facile approach to constructing controlled-release, and high-efficacy organic-inorganic hybrid nanoplatforms for tumor treatment.

## 2 Materials and methods

### 2.1 Materials

Nonionic surfactant poloxamer F127 was purchased from Yuanye Biological Technology Co., Ltd. (Shanghai, China). Trimethoxysiylpropanethiol (MPTMS) was purchased from Macklin. PTX was purchased from Tianjin Heowns Biological Technology Co., Ltd. Annexin V- fluorescein isothiocyanate (FITC)/propidium iodide (PI) double staining cell apoptosis detection kit and Live-Dead Cell Staining Kit was purchased from Keygen Biotech (Jiangsu, China). Lyso-Tracker-Green and Tubulin-Tracker Red were purchased from Shanghai Beyotime Biological Technology Co., Ltd.

### 2.2 Methods

#### 2.2.1 Synthesis of PTX@HOMNs and Nile red@HOMNs

200 mg F127 and 10 mg PTX were dissolved in ethanol, then evaporated to dryness at 45°C to obtain a micellar membrane. Then, the micellar membrane was re-dissolved in 10 mL ultra-pure water with a low-speed rotation at 45°C to obtain PTX@F127 micellar solution. MPTMS was added into PTX@F127 micellar solution in a weak alkaline environment and the solution was stirred at ambient temperature for 24 h. Then the products were subjected to dialysis against pure water to remove the unreacted supplements to obtain hybrid organosilica-micelles nanoparticles loaded with PTX (designated PTX@HOMNs). Nile red@HOMNs (designated NR@HOMNs) were prepared similarly as described above except for the use of 1.67 mg Nile red (NR).

#### 2.2.2 *In vitro* stability of PTX@HOMNs

*In vitro* stabilities of nanoparticles (NPs) were investigated by cultured NPs with deionized water, serum-free 1640 medium, PBS solution containing 10% fetal bovine serum (FBS) (pH 7.4), and glucose at 37°C for different times. The changes in particle size were measured by dynamic light scattering (DLS).

#### 2.2.3 Intermolecular interactions investigation

PTX@HOMNs were incubated with NaCl, urea, sodium lauryl sulfate (SDS), and Triton X-100 for 24 h. The changes in particle size and polydispersity index (PDI) were measured by DLS.

#### 2.2.4 Responsiveness of PTX@HOMNs and NR@HOMNs

PTX@HOMNs were suspended in PBS (0 and 10 mM dithiothreitol (DTT)). At predetermined time intervals, the solution was measured by transmission electron microscopy (TEM).

NR@HOMNs were suspended in PBS (0, 2, and 10 mM DTT). At predetermined time intervals, the solution was measured by fluorescence spectrometer.

#### 2.2.5 Cell lines and cell culture

The human ovarian cancer cell line A2780 was purchased from Keygen Biotech (Jiangsu, China). The mouse breast cancer cell line 4T1 and the mouse fibroblasts cell line L929 were donated by Prof. Xie from the Changchun Institute of Applied Chemistry, Chinese Academy of Sciences. A2780 cells and 4T1 cells were cultured in Roswell Park Memorial Institute 1640 (RMPI 1640, BI) supplemented with 10% (v/v) heat-inactivated FBS. L929 cells were cultured in Dulbecco’s modified Eagle medium (DMEM, Sigma) with 10% (v/v) FBS.

#### 2.2.6 *In vitro* cellular uptake

A2780 cells were seeded at a density of 1 × 10^5^ cells per well in six-well culture plates and incubated at 37°C. Then cells were adhered to the culture plates and treated with NR@HOMNs for 1, 3, and 5 h at 37°C in a CO_2_ incubator and 3 h at 4°C, respectively. Cells were fixed, washed, and stained with 1 mL of Hoechst 33258. The samples were observed under confocal laser scanning microscopy (CLSM). For lysosome co-localization, cells were incubated at 37°C with Lyso-Tracker Green for 30 min.

#### 2.2.7 Cell viability assays

The cytotoxicity of PTX@HOMNs in human ovarian carcinoma (A2780) and mouse breast cancer (4T1) cell lines was examined via the 3-(4,5-dimethylthiazol2-yl)-2,5-diphenyl-tetrazolium bromide (MTT) assay. Taxol and PTX@HOMNs were diluted with the fresh media in concentrations (PTX) ranging from 0.0001 to 30 µM and incubated with cells together for 48 h. Then the cells were incubated with MTT for 4 h. Dimethyl sulfoxide was added and measured at 490 nm.

#### 2.2.8 Calcein-AM/PI staining tests

To further demonstrate the antitumor efficacy of NPs, A2780 cells were costained with the calcein-AM/PI to differentiate the live/dead cells by fluorescence (green/red). Briefly, after incubating A2780 cells with PBS, Taxol, and PTX@HOMNs (in an equivalent PTX concentration of 20 μM) for 48 h, then the incubation medium was removed and calcein-AM/PI was added. Finally, the treated cells were imaged by fluorescence microscopy.

#### 2.2.9 Cell apoptosis assays

A2780 cells were seeded at a density of 1 × 10^5^ cells per well in a six-well culture plate and incubated in a CO_2_ incubator at 37°C. PTX@HOMNs and Taxol, both at 20 uM concentration, were incubated with A2780 cells for 48 h. Cells were washed, trypsinized without EDTA, and centrifuged. Cells were then suspended and stained with Annexin V-FITC and PI solution. Then, the fluorescence of the apoptotic and necrotic cells was measured by flow cytometry (FCM).

#### 2.2.10 Microtubule staining

A2780 cells were seeded in a culture plate and then treated with PBS, Taxol, and PTX@HOMNs for 24 h. After that, cells were washed gently, fixed, and washed with 0.1% Triton X-100. Then cells were incubated with an immunostaining second antibody of Tubulin-Tracker Red diluted by 0.1% Triton X-100 and 3% bull serum albumin at room temperature for 30 min in the dark. Then, the stained cells were made into slides and imaged by CLSM.

#### 2.2.11 Mice and tumor model

All animal experiments were approved by the Animal Ethics Committee of the First Hospital of Jilin University (protocol number: 20230448). Female BALB/c nude mice between 5 and 6 weeks of age were obtained from Liaoning Changsheng Biotechnology Co., Ltd. and raised under the required conditions. Mice with subcutaneous A2780 xenograft tumors were utilized as an animal modal to evaluate antitumor effects. A2780 cells were subcutaneously inoculated into the armpit of the left anterior limb of mice (5 × 10^6^ cells per 0.1 mL PBS). Then these mice were randomly divided into three groups (PBS, Taxol, and PTX@HOMNs groups, three mice of each group). PBS, Taxol, and PTX@HOMNs were intravenously injected into the corresponding mice, in which Taxol, and PTX@HOMNs at an equivalent PTX dose of 15 mg·kg^–1^ (drug weight/body weight), every other day. Mice were marked before treatment, and the body weights of mice and tumor volumes were measured before and after treatment at the designed time (0, 2, 4, 6, 8, 10 days). At the end of treatment, mice were sacrificed by cervical dislocation. The tumors were fixed and stained using hematoxylin and eosin (H&E) or TUNEL. To investigate the safety of formulations, the H&E of main organs (including heart, liver, spleen, lung, kidney) and blood routine was examined.

#### 2.2.12 Statistical analysis

All statistical analyses were performed using the student’s t-tests or one-way analysis of variance (ANOVA). Data were statistically evaluated using the Origin 2022 software (OriginLab) or GraphPad Prism 9.4.1. Statistical significant differences were defined as *p*-values <0.05 (*p < 0.05, **p < 0.01, and ***p < 0.001).

## 3 Results and discussion

### 3.1 Characterization of PTX@HOMNs

**FIGURE 1:**
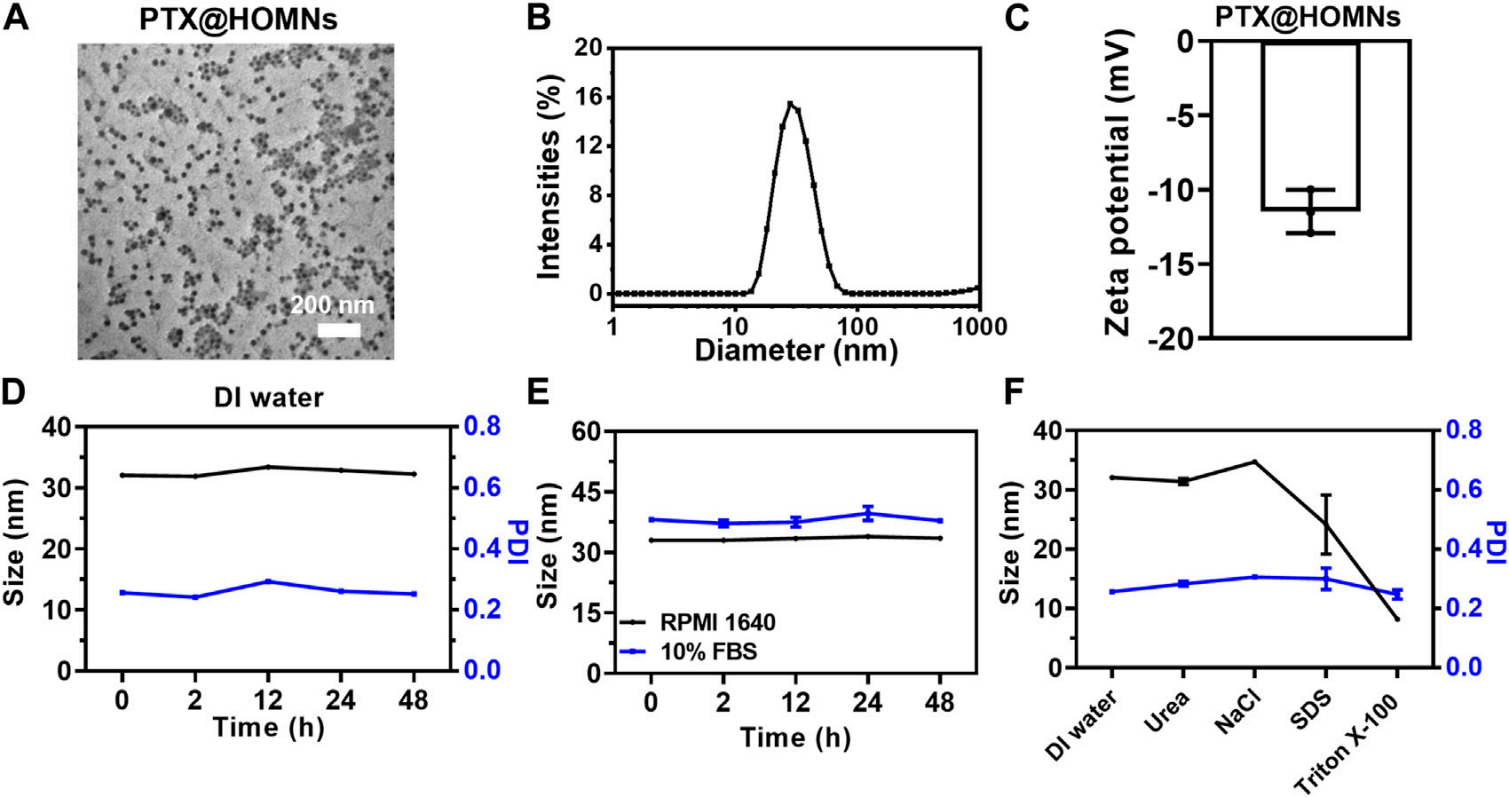
Characterization of PTX@HOMNs. **(A)** TEM images, **(B)** size distribution, and **(C)** ζ-potential of PTX@HOMNs. **(D)** Size and PDI changes of PTX@HOMNs in deionized water. **(E)** Size changes of PTX@HOMNs treated with serum-free RPMI 1640 and 10% FBS in phosphate buffer saline (PBS) for 48 h. **(F)** Size and PDI changes of PTX@HOMNs after being treated with deionized water, NaCl, urea, SDS, and Triton X-100 solutions for 24 h. Bars represent SD (*n* = 3).

MPTMS was added to micellar solution, and hydrolyzed to produce disulfide bonds in the confined hydrophobic regions of PPO blocks in F127 micelles. The PTX@HOMNs exhibited a uniform spherical morphology as shown by TEM (Figure 1A). The DLS was employed to investigate the hydrodynamic sizes of PTX@HOMNs and HOMNs, which were 32.07 ± 0.13 nm and 19.46 ± 0.28 nm, as determined by DLS (Figure 1B; Supplementary Figure S1A). PTX@HOMNs possessed more negative ζ-potential of −11.46 ± 1.19 mV, compared with −5.94 ± 0.97 mV for HOMNs (Figure 1C; Supplementary Figure S1B). We speculate that the reduced zeta potential corresponding to the large size of PTX@HOMNs may be due to more exposure to negative-charged on the surface with silicone hydroxyl and sulfhydryl groups after the incorporation of paclitaxel. The drug-loading content and drug-loading efficiency of PTX are 1.4% and 22%. PTX@HOMNs in deionized water, serum-free RPMI 1640 medium, and PBS solution containing 10% FBS, and 5% glucose showed negligible size changes (Figures 1D, E; Supplementary Figure S2), suggesting that confinement of PTX via disulfide-bond linked silica promoted the colloidal stability. To investigate the coassembly mechanism of PTX@HOMNs, we further incubated PTX@HOMNs with urea, NaCl, SDS, and Triton X-100 for 24 h. The particle size of PTX@HOMNs decreased when co-incubated with Triton X-100, demonstrating that in the assembly progress, hydrophobic interactions played a dominant role (Figure 1F).

### 3.2 Reduction-responsive drug release

**FIGURE 2:**
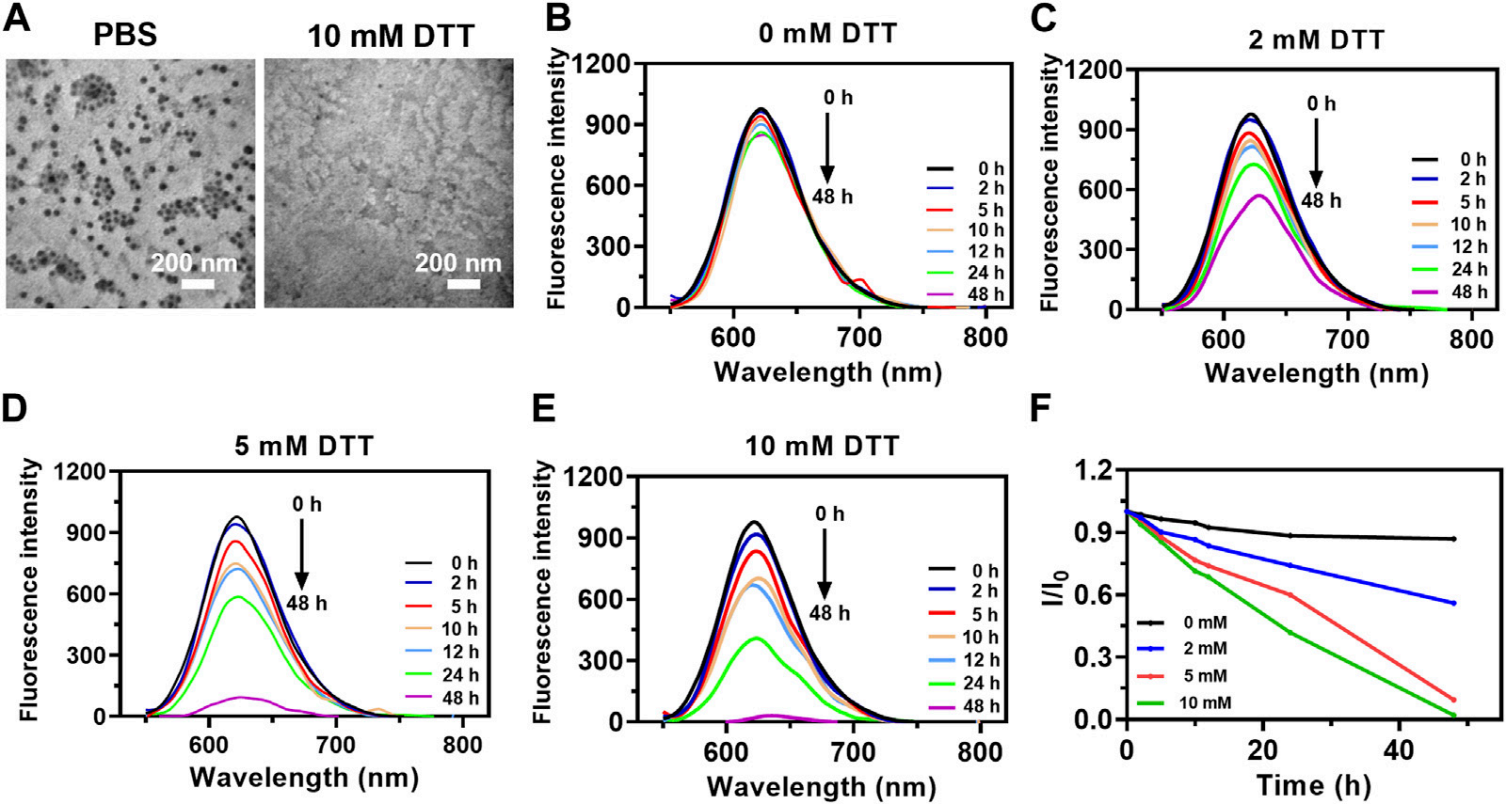
Reduction responsiveness of PTX@HOMNs and NR@HOMNs. **(A)** TEM images of PTX@HOMNs after treatment with 0 or 10 mM DTT. Fluorescence intensity changes of NR@HOMNs after treatment with 0 **(B)**, 2 **(C)**, 5 **(D)**, and 10 **(E)** mM DTT. **(F)** The relative changes in the emission of NR@HOMNs after treatment with 0, 2, 5, and 10 mM DTT. I and I_0_ are the FL intensities of NR@HOMNs at 622 nm in the presence and absence of DTT, respectively.

Disulfide bond-doped cores in the F127 micelles were formed via the oxidation of partial sulfhydryl groups of MPTMS by O_2_ in the air during the hydrolysis/condensation reaction (Niu et al., 2021). To investigate the reduction-responsive drug release characteristic, the morphologies of nanoparticles mixed with different concentrations of DTT were monitored. After incubation with 10 mM DTT, PTX@HOMNs showed a significant morphological transition (Figure 2A). As a fluorescent model, Nile Red instead of paclitaxel was encapsulated into nanocarriers with the same method. The fluorescence intensity of NR@HOMNs remained almost unchanged (Figure 2B). Significant decreases in the fluorescence intensity of NR@HOMNs after mixing with 2, 5, and 10 mM DTT (Figures 2C–E). Concentration-dependent fluorescence attenuation confirmed the reduction responsiveness of NR@HOMNs (Figure 2F). The HOMNs displayed the ability to degradable and regulate drug release via changing the amount of DTT.

### 3.3 Cellular uptake of PTX@HOMNs

**FIGURE 3:**
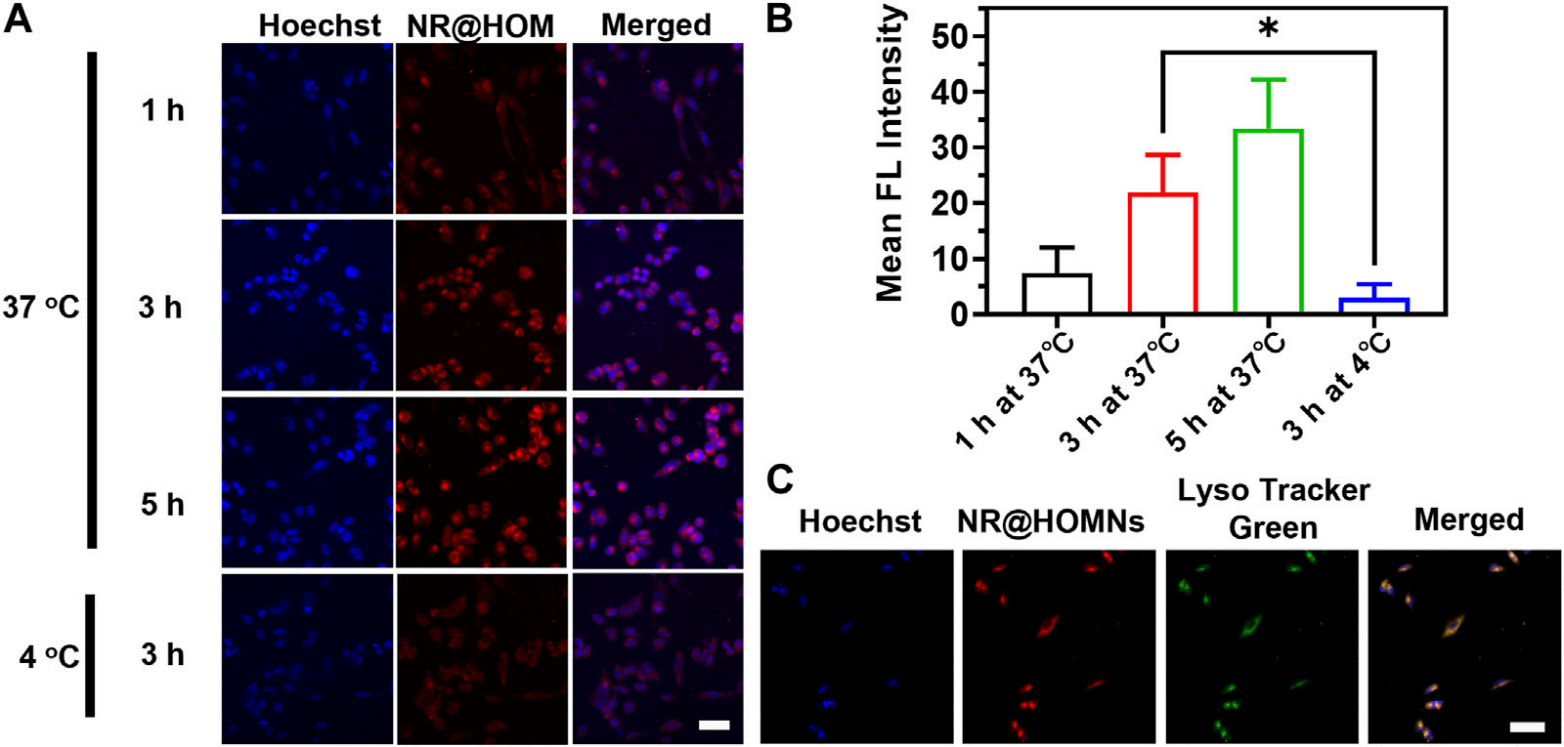
**(A)** CLSM images and **(B)** the mean fluorescence intensity of A2780 cells treated with NR@HOMNs for 1, 3, and 5 h at 37 °C, and 3 h at 4 °C. **(C)** CLSM images of A2780 cells incubated with NR@HOMNs for 5 h and stained with Lyso-Tracker Green. Scale bars, 40 µm.

Since paclitaxel does not have fluorescence, Nile red was used to replace paclitaxel wrapped in the frame for visualizing cellular internalization and distribution. A2780 cells were incubated with NR@HOMNs at different times at 37 °C and 4 °C. A time-dependent increase of the intracellular red fluorescence intensity from 1 to 5 h in the cytoplasm of A2780 cells was observed by CLSM, demonstrating that the organosilica-micellar hybrid nanoplatforms could be efficiently internalized by cells and endocytosis was time-dependent (Figures 3A, B). Besides, the different incubating temperatures showed the endocytosis of A2780 cells was energy-dependent (Figures 3A, B). The red fluorescence of NR@HOMNs was perfectly colocalized with the green fluorescence of Lyso Tracker, revealing the accumulation of NR@HOMNs in the lysosome (Figure 3C).

### 3.4 Cytotoxicity of PTX@HOMNs

**FIGURE 4:**
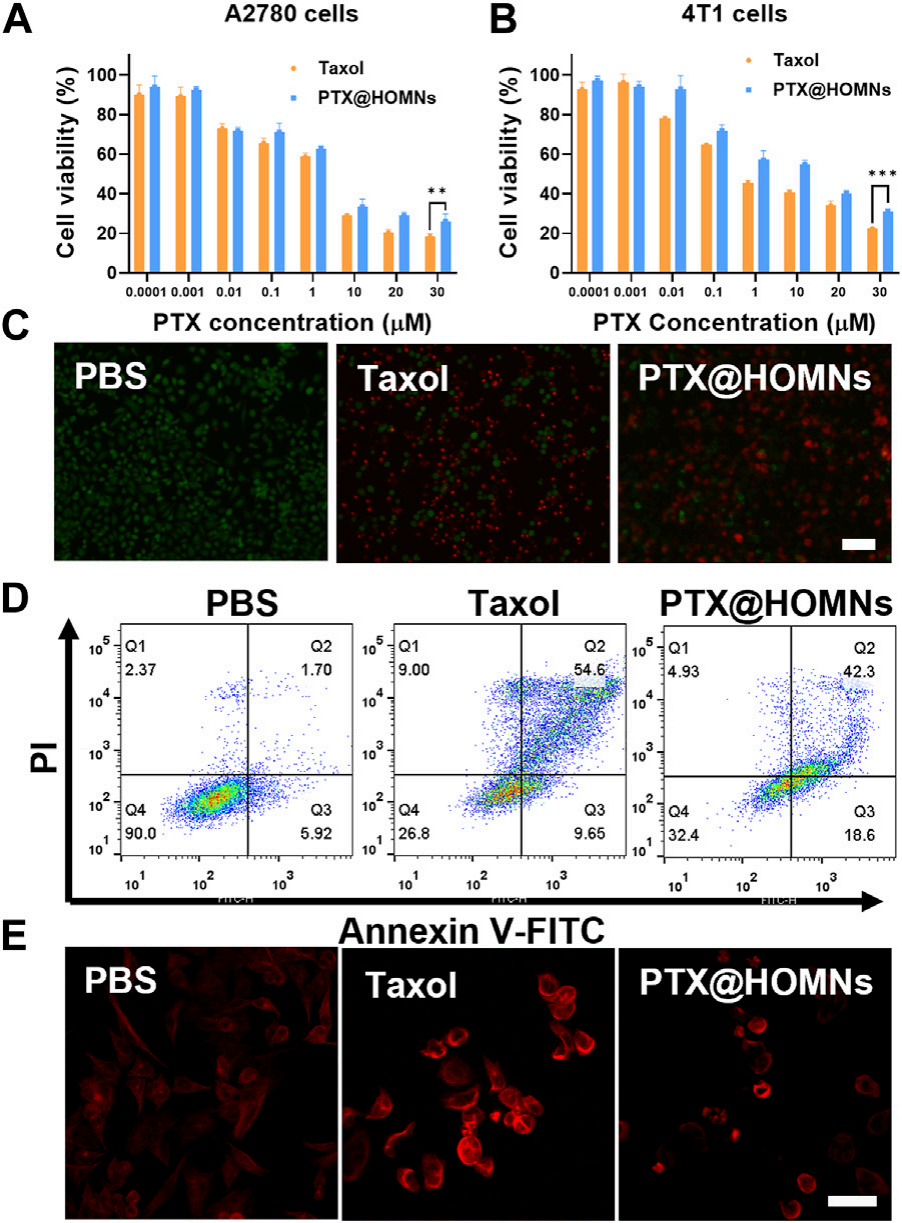
Cytotoxicity of PTX@HOMNs in cells. Cell viability incubated with Taxol and PTX@HOMNs toward A2780 **(A)** and 4T1 **(B)** cells after incubation in the concentrations varied from 0.0001 to 30 μM for 48 h via MTT assays (*n* = 3). **(C)** Fluorescence images of A2780 cells, co-stained with calcein-AM/PI after incubation with PBS, Taxol, and PTX@HOMNs for 48 h. Scale bars, 100 μm. **(D)** Apoptosis of A2780 cells after incubation with PBS, Taxol, and PTX@HOMNs for 48 h by FCM. **(E)** CLSM images of microtubules in A2780 cells after incubation with PBS, Taxol, and PTX@HOMNs for 24 h as determined by CLSM. Scale bars, 40 μm.

We further evaluated the *in vitro* cytotoxicity of PTX@HOMNs through MTT assays. A2780 and 4T1 cells were incubated with Taxol and PTX@HOMNs, respectively. As illustrated in Figures 4A, B, PTX@HOMNs displayed comparable cancer cell-killing efficacy with Taxol. The half-maximal inhibitory concentration (IC50) values of Taxol and PTX@HOMNs were 0.6751ug/mL and 1.445ug/mL, respectively. Compared with Taxol, the cytotoxicity of PTX@HOMNs against L929 cells was significantly weaker (Supplementary Figure S3A). HOMNs exhibited good biocompatibility toward L929, A2780, and 4T1 cells (Supplementary Figures S3B–S3D). Calcein-AM/PI staining tests were conducted to evaluate the therapeutic effect of PTX@HOMNs. A2780 cells showed much more red fluorescence than the PBS group after incubation with Taxol and PTX@HOMNs (Figure 4C). Then, the apoptosis effect of PTX@HOMNs on A2780 cells was quantitatively evaluated by FCM with annexin-V FITC and PI. PTX@HOMNs exhibited slightly total apoptotic ratios (Q2 + Q3) of 61.9%, indicating the potent antitumor effect via apoptotic pathway (Figure 4D). Tubulin immunostaining assays showed that the microtubule network of A2780 cells in the PBS group was uniformly distributed in the cytoplasm, while the Taxol and PTX@HOMNs groups showed various degrees of newly formed microtubule bundles or aggregation (Figure 4E).

### 3.5 *In vivo* antitumor efficacy and biosafety

**FIGURE 5:**
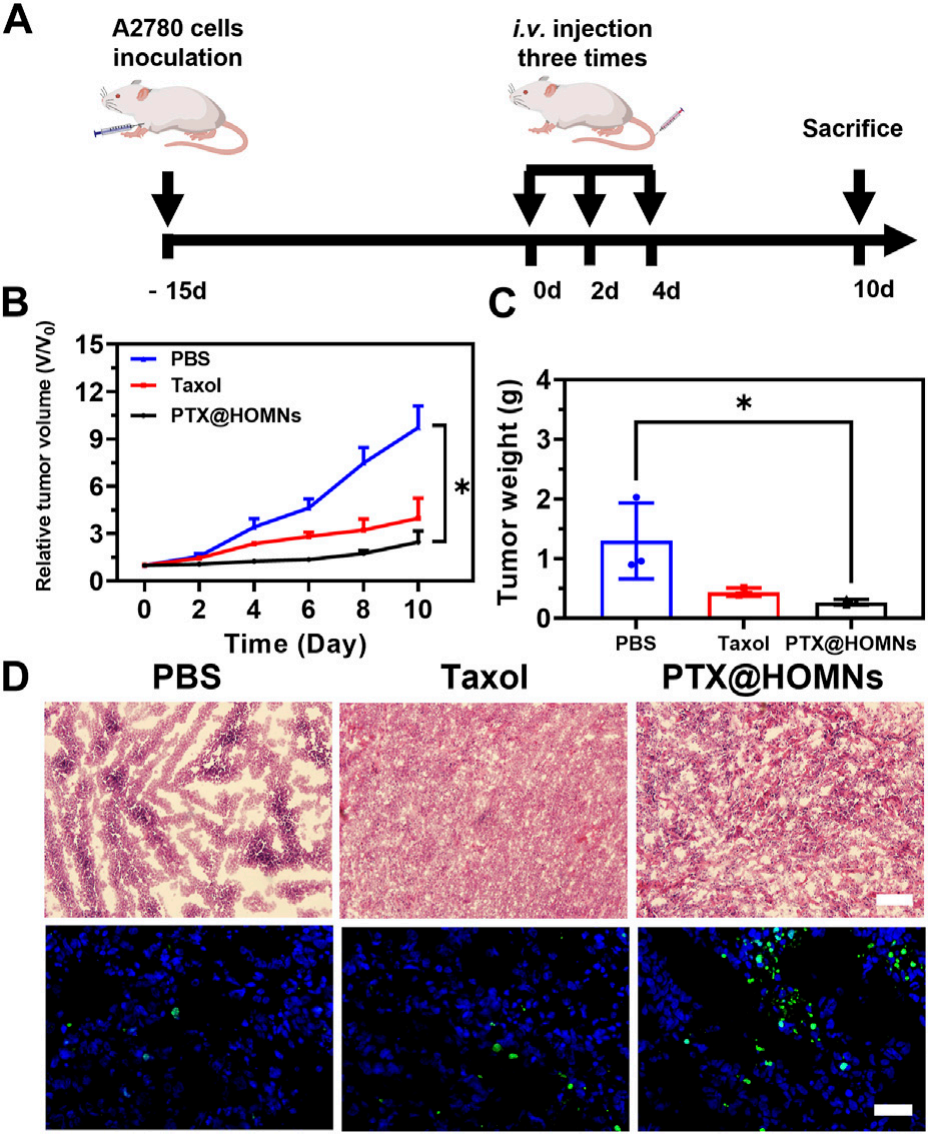
*In vivo* antitumor efficacy of PTX@HOMNs. **(A)** Schematic representation of the animal experiment treatment. Administrated with PBS, Taxol, and PTX@HOMNs. **(B)** Growth curve of tumor volumes of A2780-bearing mice after being treated with PBS, Taxol, and PTX@HOMNs. **(C)** Weights of excised tumors of PBS, Taxol, and PTX@HOMNs. **(D)** H&E (upper) and TUNEL (lower) staining images of tumor tissues collected from A2780 tumor-bearing mice administrated with PBS, Taxol, and PTX@HOMNs. Scale bar, 200 μm.

To evaluate the *in vivo* antitumor efficacy of PTX@HOMN, a subcutaneous A2780 human ovarian tumor model was established. The animal experiment was followed by the protocol illustrated in Figure 5A. When the tumor volume reached approximately 170–220 mm^3^, the tumor-bearing mice were randomly divided into three groups: PBS, Taxol, and PTX@HOMNs. The tumors of mice in the PBS group grew faster, while those of the PTX@HOMNs and Taxol groups showed smaller tumor sizes and lighter tumor weights (Figures 5B, C). The images of excised tumors showed smaller tumor size in PTX@HOMNs, which further confirmed the good tumor inhibition capability of PTX@HOMNs (Supplementary Figure S4). H&E and TUNEL staining assays showed more significant cancer cell damage and apoptosis in the PTX@HOMNs group than that in the PBS group (Figure 5D).

**FIGURE 6:**
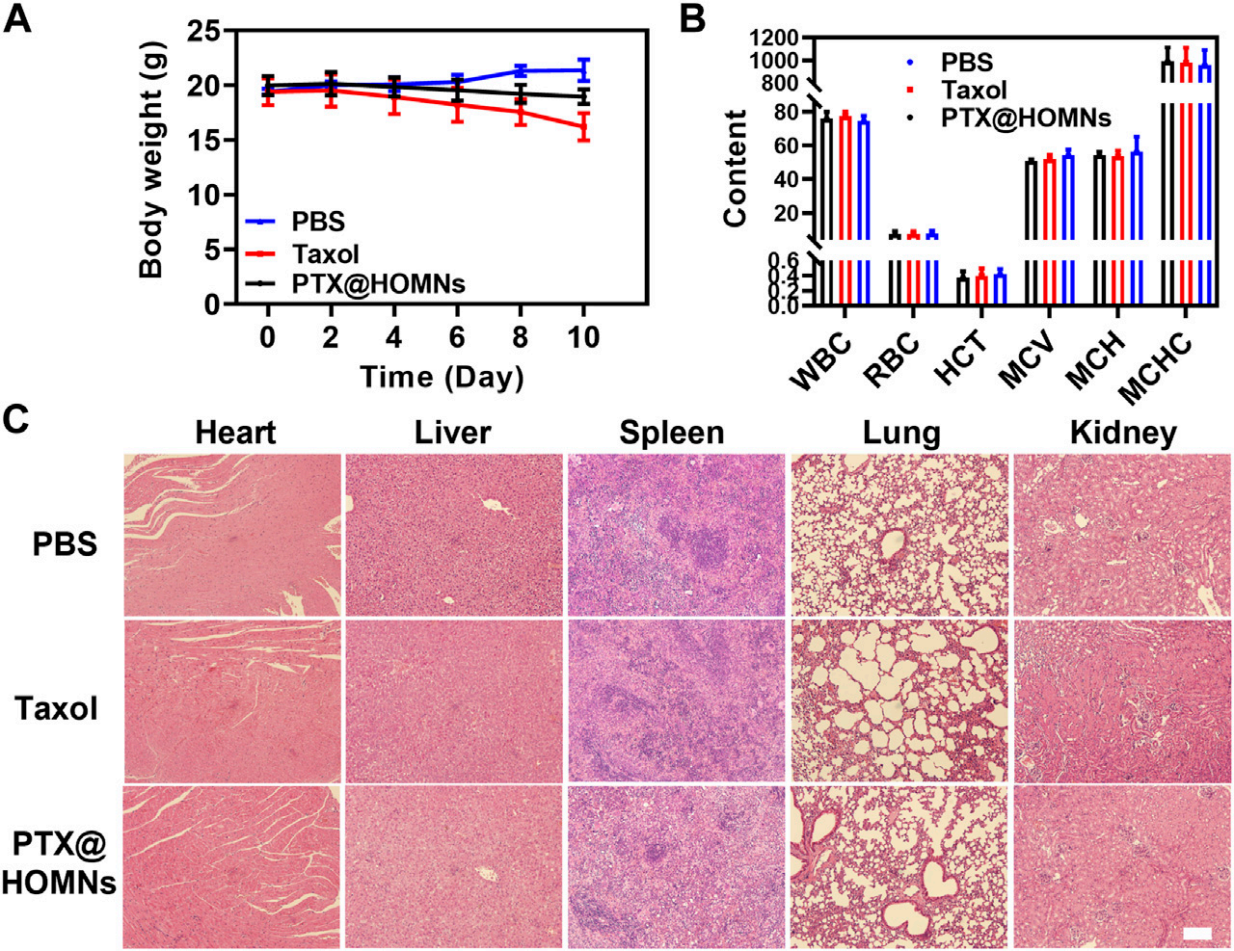
**(A)** Body weight changes in mice in PBS, Taxol, and PTX@HOMNs groups during treatment. **(B)** Routine blood analysis of mice in PBS, Taxol, and PTX@HOMNs groups. Bars express SD (*n* = 3). **(C)** H&E staining images of major organs (heart, liver, spleen, lung, and kidney) in different groups. Scale bar, 200 μm.

The systemic toxicity of Taxol and PTX@HOMNs was estimated. The body weights of mice in the Taxol group showed obvious weight loss during the treatment. While PTX@HOMNs showed a negligible change in body weight, verifying their reduced systemic toxicity (Figure 6A). The blood routine analysis showed no obvious hematopoietic dysfunction in the three groups (Figure 6B). In addition, no detectable pathological change was observed in images of H&E-stained major organs (Figure 6C). These results suggested that the PTX@HOMNs had good biosafety *in vivo*.

## 4 Conclusion

In summary, our work shows the fabrication of disulfide bond-based organosilica-micellar hybrid nanoplatforms with specific reduction-triggered drug release. In addition, the as-fabricated PTX@HOMNs could remain stable in the physiological microenvironment. PTX@HOMNs could respond to DTT and release PTX *in vitro*. PTX@HOMNs exhibited selective cytotoxicity towards tumor cells. PTX@HOMNs showed superior antitumor activity *in vivo* and alleviated system toxicity. This work provides a promising method to develop controllable organosilica-micellar hybrid nanoparticles for tumor-specific chemotherapy.

## Data Availability

The original contributions presented in the study are included in the article/Supplementary Material, further inquiries can be directed to the corresponding author.
