# Supplementary material for: Reduction-responsive supramolecular hybridized paclitaxel nanoparticles for tumor treatment
Source: Front Bioeng Biotechnol. 2023 Sep 1;11:1257788. doi: 10.3389/fbioe.2023.1257788 (PMC10505395; doi:10.3389/fbioe.2023.1257788)
Supplement: Supplementary file 1 [file DataSheet1.docx]

Supplementary Material

Yuhan Wang^1^, Yingli Cui^1^, Tinggeng Dai^1^, Ying Yue^1*^

^1^Department of Gynecological Oncology, The First Hospital of Jilin University, Xinmin Street, Changchun, Jilin 130021, P. R. China

*** Correspondence:**Ying Yue
yuey@jlu.edu.cn


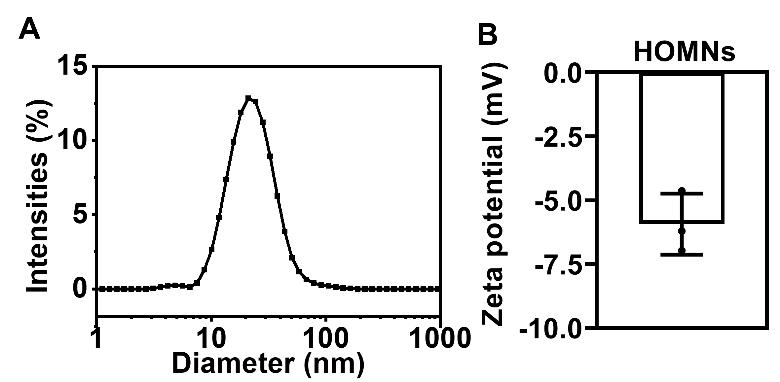


**Figure S1.** (A) Size distribution of HOMNs. (B) ζ-potential of HOMNs.


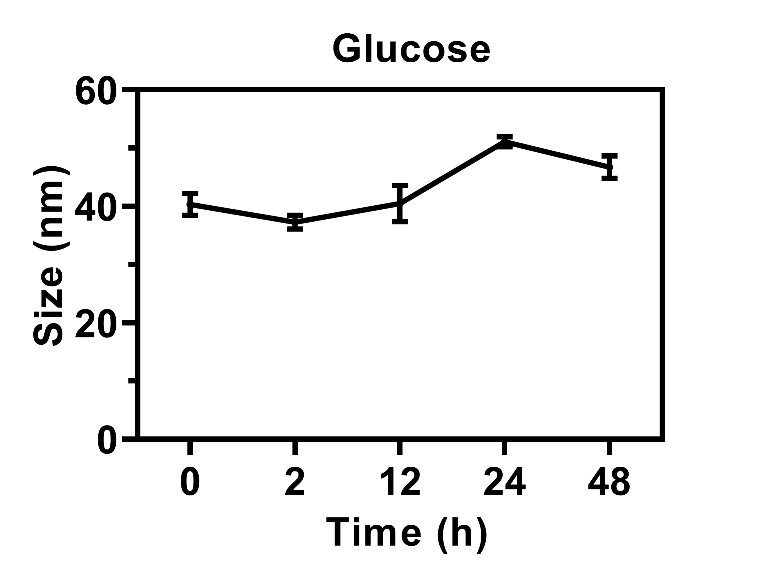


**Figure S2.** Size changes of PTX@HOMNs treated with glucose for 48 h. Bars represent SD (n = 3).


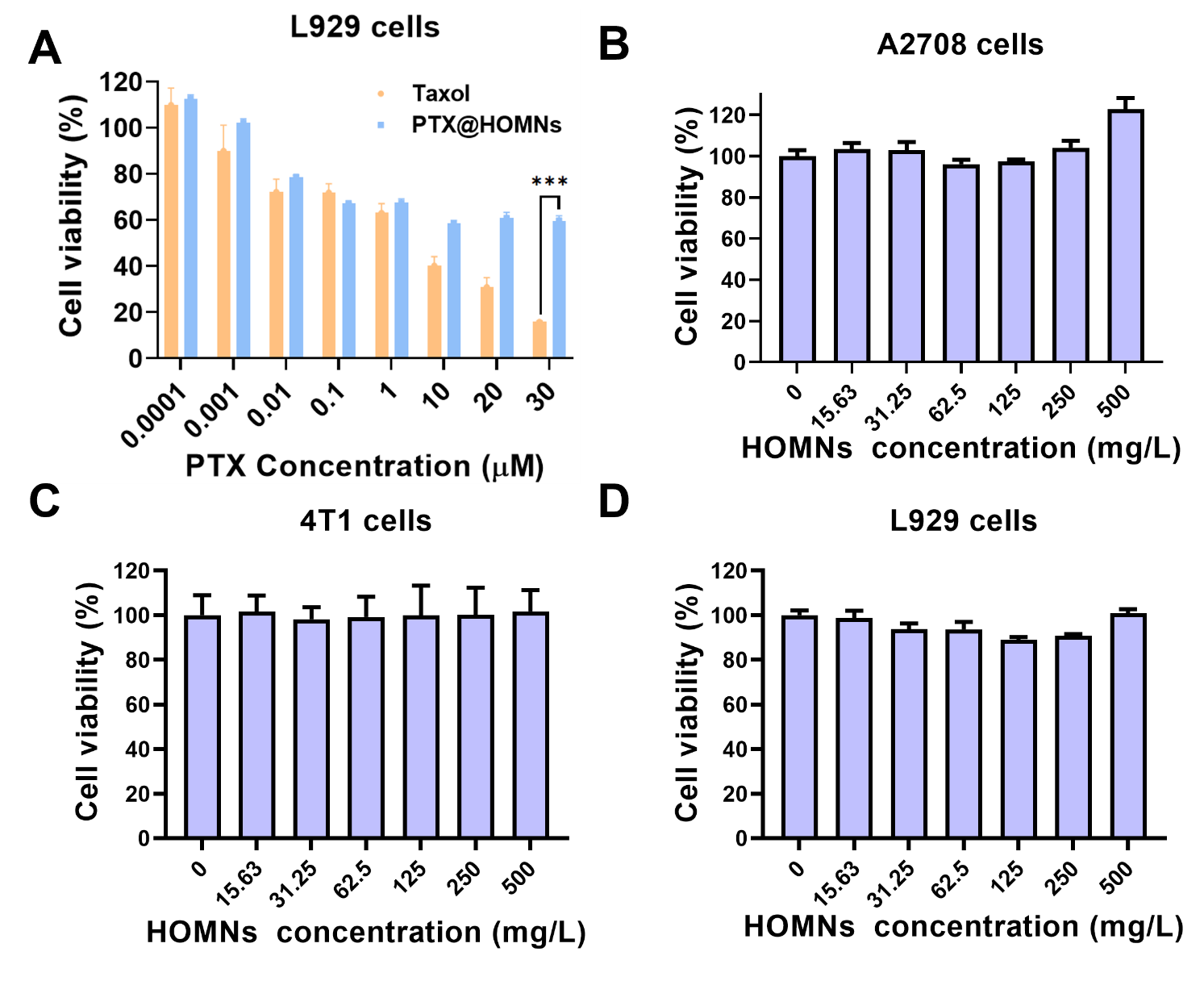


**Figure S3.** (A) L929 cells incubated with PTX@HOMNs for 48 h in different concentrations by MTT assay. (B) Viabilities of A2780 cells incubated with HOMNs for 48 h by MTT assay. (C) Viabilities of 4T1 cells incubated with HOMNs for 48 h by MTT assay. (D) Viabilities of L929 cells incubated with HOMNs for 48 h by MTT assay.


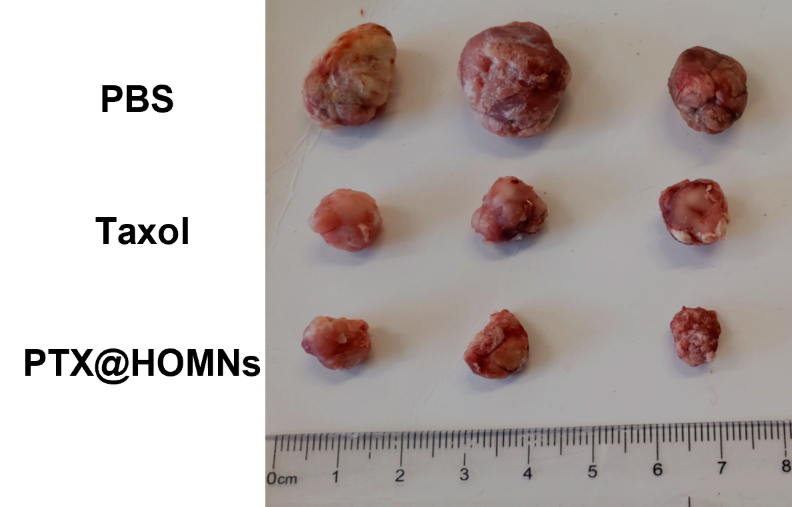


**Figure S4.** The tumors excised from the A2780-bearing mice after the treatments by PBS, Taxol, and PTX@HOMNs.
